# Fertility Preservation in BRCA1/2 Germline Mutation Carriers: An Overview

**DOI:** 10.3390/life14050615

**Published:** 2024-05-10

**Authors:** Erica Silvestris, Gennaro Cormio, Vera Loizzi, Giacomo Corrado, Francesca Arezzo, Easter Anna Petracca

**Affiliations:** 1 Gynecologic Oncology Unit, IRCCS Istituto Tumori “Giovanni Paolo II”, 70124 Bari, Italy; gennaro.cormio@uniba.it (G.C.); vera.loizzi@uniba.it (V.L.); francesca.arezzo@uniba.it (F.A.); e.petracca@oncologico.bari.it (E.A.P.); 2 Department of Interdisciplinary Medicine (DIM), University of Bari “Aldo Moro”, 70121 Bari, Italy; 3 Department of Woman, Child Health and Public Health, Gynecologic Oncology Unit, Fondazione Policlinico Universitario A. Gemelli, IRCCS, 00136 Rome, Italy; giacomo.corrado@policlinicogemelli.it

**Keywords:** BRCA, fertility preservation, oncofertility, ovarian stem cell, premature ovarian failure

## Abstract

BRCA1 and BRCA2 mutations are responsible for a higher incidence of breast and ovarian cancer (from 55% up to 70% vs. 12% in the general population). If their functions have been widely investigated in the onset of these malignancies, still little is known about their role in fertility impairment. Cancer patients treated with antineoplastic drugs can be susceptible to their gonadotoxicity and, in women, some of them can induce apoptotic program in premature ovarian follicles, progressive depletion of ovarian reserve and, consequently, cancer treatment-related infertility (CTRI). BRCA variants seem to be associated with early infertility, thus accelerating treatment impairment of ovaries and making women face the concrete possibility of an early pregnancy. In this regard, fertility preservation (FP) procedures should be discussed in oncofertility counseling—from the first line of prevention with risk-reducing salpingo-oophorectomy (RRSO) to the new experimental ovarian stem cells (OSCs) model as a new way to obtain in vitro-differentiated oocytes, several techniques may represent a valid option to BRCA-mutated patients. In this review, we revisit knowledge about BRCA involvement in lower fertility, pregnancy feasibility, and the fertility preservation (FP) options available.

## 1. Introduction

*BRCA1* and *BRCA2* (BReast CAncer susceptibility genes 1 and 2) are tumor suppressor genes located on chromosome 17q21 and 13q12, respectively, and primarily known for their involvement in the repair of double-strand breaks (DSBs) of DNA, but also in chromatin remodeling [1], telomere preservation [2] and embryogenesis [3]. *BRCA1/2* loss of function (LOF) mutations take to genomic instability, cell cycle arrest and apoptosis [4]. More than 3500 variants have been identified and distinguished into two types, somatic (non-inheritable) or germline (*BRCA*_germ_), which are instead transmitted to offspring in an autosomal dominant way. Each first-degree relative affected by the mutation has a 50% chance of also being a carrier of the mutated gene. *BRCA*_germ_ are responsible for an increased risk of breast or ovarian cancer (HBOC—hereditary breast and ovarian cancer) development, which together represent 5–7% of all cancer cases worldwide [5]. The risk of breast cancer (BC) development in the general population has been estimated at around 12%, compared to *BRCA* variants carriers, which rises to 55–70%. Similarly, for ovarian cancer (OC), the risk of development in healthy individuals is estimated to be 1–2%, while in *BRCA2*-positive individuals, it increases to 39–44%, with a greater impact for *BRCA1* variants (11–17%) [6].

The prevalence of *BRCA* variants has been estimated from 1:400 to 1:500 [7]. It seems that both *BRCA*-related BC and OC risk increases with patient age and with previous familiar history [8]. Therefore, it is essential to identify and screen individuals with *BRCA* variants, as the earlier the detection, the higher the chances of successful treatment.

Interestingly, some populations are associated with higher prevalence due to founder mutations; Ashkenazi Jewish have a 1 in 40 chance of carrying a *BRCA* mutation [9], while the Ammassalik population from Greenland has a prevalence of 1 in 10 to 1 in 100 [10].

*BRCA*_germ_ not only increases the risk of BC and OC but also makes individuals more susceptible to pancreatic [11] and prostate cancer [12], eye melanomas [13] and others [5]. However, these genes show incomplete penetrance, so disease onset and severity are difficult to predict. Additionally, subjects who carry *BRCA* variants but do not develop cancer can be identified as “previvors” [14].

Since HBOCs are hormone-sensitive neoplasms [15,16], and *BRCA* variants are triggering factors for their development, in previous years, scientific interest has focused on finding possible correlations between *BRCA* alterations and fertility injury in young *BRCA*-mutated women. Several studies have confirmed that variant occurrence is related to lower fertility, with strong consequences on women’s life planning. Thus, in this report, our aim is to summarise recent data concerning fertility impairment in *BRCA*-mutated patients, pregnancy likelihood and fertility options.

## 2. The Investigation of *BRCA*-Mutated Carriers

More than 80% of invasive BC in individuals with *BRCA* mutations are ductal carcinomas, while 2–8% are lobular carcinomas. Medullary BC, rarely diagnosed in sporadic cases (less than 1%), can be found in up to 19% of those with BRCA1 mutations. Additionally, there is a 63% risk of developing a second primary contralateral breast cancer within 25 years of the initial diagnosis for carriers affected with *BRCA* variants, with *BRCA1* carriers having the highest risk [17].

The National Comprehensive Cancer Network (NCCN) recommends a surveillance plan for mutated women, starting with awareness from the age of 18, clinical breast examinations every 6–12 months from 25 and annual breast MR imaging or mammography at 25–29 yrs. From 30–75 yrs, women should undergo annual mammography combined with breast MR imaging. With the high *BRCA*-related BC growth rate, alternating MR imaging and mammographic screening exams every 6 months have proven to be clinically effective [18]. To significantly reduce the risk of BC development, patients may opt for prophylactic mastectomy, which can lower the risk by at least 90%. According to the National Cancer Data Base, the rate of contralateral prophylactic mastectomy is 9.7% for all age groups, and this percentage rises to 26% for <45 women [19]. However, the decision to undergo mastectomy is influenced by other factors rather than *BRCA* mutation, such as the patient’s childbearing planning or the age of onset in the youngest affected family member.

Chemoprevention with tamoxifen can be an alternative option, but extensive evaluation has revealed significant side effects such as endometrial abnormality, blood clots and higher incidence of endometrial cancer in postmenopausal women, which significantly contribute to morbidity and mortality. Endometrial thickness increase is evaluated through transvaginal ultrasonography, but the cutoff value in women undergoing tamoxifen or post-tamoxifen therapy is still debated. Drug modifications showed raloxifene as a safer alternative for patients with no impact on therapeutic effects [20].

Among imaging tools, mammography has only 30% of the sensitivity for detecting breast carcinoma in *BRCA1/2*-mutated carriers (*BRCA*^mut^) compared to 83% estimated sensitivity in the general population [21,22]. In addition, almost half of the women in this group are diagnosed with BC less than a year after receiving normal mammography results. Lower mammography sensitivity can partially be related to breast tissue density that hides the tumoral area. Thus, for younger women with more dense breast tissue, digital mammography is more effective in detection. Furthermore, *BRCA*-associated breast malignancies often display imaging features as well as benign lesions, resulting in mammography false-negative results.

Breast MR imaging has shown to be highly effective, with up to 100% sensitivity and 97% specificity, enhancing the possibility of detecting BC at early stages, especially for *BRCA*^mut^ [23]. Such performances have been confirmed by a screening trial of Passaperuma K., involving 500 *BRCA* mutation carriers, in which MR imaging showed a sensitivity of 94%, compared to only 9% shown by mammography [24].

Supplemental screening with ultrasound (US) provides no additional benefit compared to mammography and MR imaging. However, breast US can be useful to bridge the relatively long time interval between the annual surveillance rounds or can be an alternative tool for high-risk women who cannot undergo MR imaging [23].

Next to US, elastography can assess breast tumors according to their stiffness, which is directly linked to the risk of malignancy. Two different elastographic techniques are used to assess tumor stiffness: strain elastography and shear-wave speed elastography (SWE). The former is a qualitative method that compares the strain of the tumor to that of the surrounding tissue. SWE gives absolute measures of tumor stiffness. Strain is inversely proportional to tissue stiffness and is displayed as a color-coded overlay on US imaging [25]. However, elastography is more specific but less sensitive than US [26], although it is simple to use and understand, making it useful for assessing breast tumors, especially for less experienced operators. Despite this, there is currently no established optimal diagnostic approach for combining elastography with US imaging.

Recently, OC has been grouped into two types based on its features and precursor lesions. Type 1 includes low-grade serous, low-grade endometrioid, clear cell, and mucinous histology, which are less common and lethal compared to type 2 carcinomas [27]. *BRCA* genetic mutations are instead associated with type 2 carcinomas, with more than 90% of *BRCA*-associated OC being high-grade serous adenocarcinomas [28]. Type 2 also includes high-grade serous, high-grade endometrioid, undifferentiated carcinomas, and carcinosarcomas, accounting for most epithelial cancer deaths [27].

OC is also linked to other hereditary cancer syndromes, such as Lynch syndrome (LS), also known as hereditary nonpolyposis colorectal cancer. LS is a hereditary condition with an increased risk of colorectal cancer development. Additionally, there is also a higher likelihood of endometrial and ovarian cancer development, respectively 30–71% and 4–24%, which increases in the case of survived women with LS [29]. LS is caused by mutations in DNA mismatch repair genes, which lead to microsatellite instability, where extra nucleotides are inserted in microsatellite repeats and affect the regulation of cell proliferation and cell cycle. LS has been identified especially in women with the clear cell and endometrioid subtypes of OC. Hence, for patients with endometrioid or clear cell histology or those with a significant personal or family history of LS-related cancers, like endometrial and colorectal cancer, LS and *BRCA1/2* genetic testing should be considered [30].

It is universally recognized that the best choice to prevent OC is recurring RRSO. RRSO entails surgical laparoscopic removal of both ovaries and fallopian tubes while still free from any abnormalities, with microscopic examination of sections for occult cancer research. Since *BRCA1*-related OC is diagnosed at a median age of 54 y/o, it is recommended that women with *BRCA1* mutations should undergo RRSO from the age of 35–40 yrs. Instead, the *BRCA2* median age of diagnosis is 59.5 y/o, and the risk of OC development before menopause is lower with this mutation (less than 3% of cases; hence, prophylactic surgery in *BRCA2*-positive patients can be delayed at 40–45 yrs [31,32].

Surgical oophorectomy reduces the risk of OC development by at least 85% for *BRCA*^mut^. Furthermore, those patients who undergo RRSO before menopause may also experience a 50% reduction in the risk of breast cancer [28]. Nevertheless, it should not be performed before completing childbearing, if possible.

The early detection of OC can be improved with a surveillance schedule consisting of periodic transvaginal US evaluation, periodic serum CA-125 level assessment and clinical examination every 6–12 months. These procedures seem to be particularly beneficial when started 30–35 yrs or 10 years prior to the earliest diagnosis in the family [28].

NCCN guidelines advise genetic counseling for all patients with invasive non-mucinous epithelial ovarian carcinomas, regardless of their family history of BC or OC [18].

Therefore, although it is crucial to highlight as a prophylactic that mastectomy and RRSO significantly decrease the risk of cancer, their negative physical and psychological effects should not be underestimated.

## 3. *BRCA* Mutations and Lower Fertility

As already described, *BRCA* gene LOF mutations force cells to employ a non-homologous and non-conservative pathway, more prone to errors, to deal with DNA damage. Fixed errors can be transmitted with replication unless apoptotic programs stop mutations from spreading with cell death. This system becomes particularly dangerous in slowly dividing or non-dividing cells, such as in ovaries, where apoptosis means loss of oocytes and premature ovarian failure (POF) [33,34]. In this regard, several studies demonstrated that the *BRCA*^mut^ shows a decreased number of oocytes, while there are still conflicting opinions concerning ovarian quality impairment. In contrast, innovative theories claim increased fertility in *BRCA*^mut^, disputing that if *BRCA* mutations are inherited through generations, it could only be due to an evolutionary advantage, translated into increased fertility [35,36].

Furthermore, recent studies have shown higher *BRCA*^mut^ ovary susceptibility to fibrosis compared to age-matched healthy ones. This preliminary result could consolidate evidence of POF development in carriers and *BRCA1/2* roles in cancer progression [37].

Since the anti-Mullerian hormone is an important marker for ovarian reserve (OR) evaluation, its tendency has been investigated in *BRCA*^mut^ women, in which decreased levels have been found in comparison with non-mutated controls (with BRCA1 accounting for a 25% decrease [38] and BRCA2 accounting for 33% [32]). Other studies found instead no differences between carriers and non-carriers [39,40]. Furthermore, *BRCA1* seems to have a worse impact with age since AMH concentrations are up to 10-fold lower in >35 women compared with younger carriers [41,42]. In agreement, pregnancy should not be delayed beyond 35 years of age [34].

As demonstrated, *BRCA*^mut^ are overexposed to chemotherapy (CT)-induced amenorrhea because of their lower OR condition. In a study conducted by Valentini A. et al., a correlation between amenorrhea occurrence and patient’s age has been found. The probability of experiencing amenorrhea ranged from 7.2% in under 30-year-old *BRCA*^mut^ patients to 33% in women between 31 and 44 yrs and 79% in over 45-year-old carriers [43]. In this study, amenorrhea was defined as the loss of menstrual cycles for more than two years, starting within two years from the first chemotherapy cycle, with no resumption. It is noteworthy that individuals with *BRCA* mutations tend to reach natural menopause approximately three years earlier than healthy women but without evidence of a significant impact on fertility [44].

As indirect proof that early menopause in *BRCA*^mut^ is due to *BRCA* gene LOF, it is interesting that even patients affected by Fanconi Anemia are affected by reduced fertility and premature menopause [45]. Fanconi Anemia is, in fact, related to different DNA repair gene mutations, known as FANC genes, with FANCS and FANCD1 corresponding, respectively, to *BRCA1* and *BRCA2*.

Moreover, estrogen production has also been suggested to promote carcinogenesis, together with gonadotropins increased release, linked to premature menopause: hormonal imbalance could explain abnormal cell growth in ovaries and ovarian cancer development [46].

## 4. Pregnancy in *BRCA* Carriers

**Figure 1:**
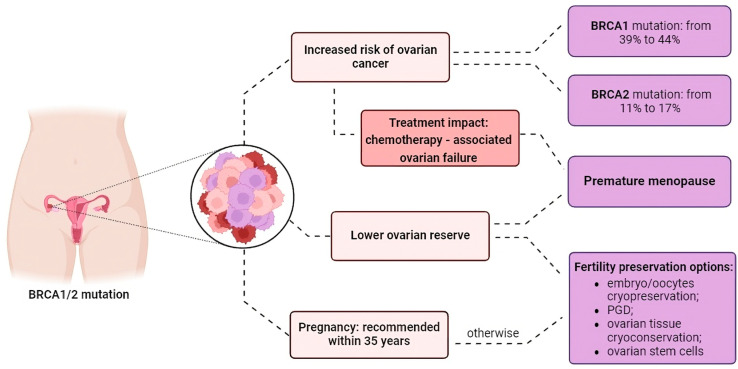
Main features characterizing *BRCA*-mutated women.

As stated above, the risk of cancer development during reproductive age in the presence of *BRCA* variants is higher compared to non-carriers [6]. Therefore, it is extremely challenging to plan pregnancy when young and still healthy patients must deal with prophylactic mastectomy and/or risk-reducing salpingo-oophorectomy (RRSO), chemotherapy-related POF and the dilemma of variant transmission to offspring. Hence, healthcare providers should recommend fulfilling their childbearing desire in youth, if possible [47], as reported in Figure 1. This is especially important since the risk of cancer development and ovarian impairment tend to increase with age [43].

According to the Society of Obstetricians and Gynaecologists of Canada (SOGC), when tumor diagnosis occurs, women should wait at least 3 years before considering pregnancy, but 5 years if lymph nodes are also involved [48].

If the diagnosis occurs during pregnancy or within 1 year of giving birth, we talk about “pregnancy-related cancer”, which can lead to treatment procrastination or hormonal negative impact on cancer metastatic behavior. Sometimes, pregnancy can even delay cancer diagnosis; hence, pregnant cancer patients show a lower survival rate with respect to oncological patients without pregnancy [49].

In the assessment of pregnancy and cancer correlation, controversial assumptions have been declared, with most of the focus on BC patients. Despite the significantly higher risk of cesarean section, lower birth weight and preterm birth with respect to the general population, BC survivors with a pregnancy show increased disease-free and overall survival than BC survivors who have not been pregnant (0.89 to 0.49 and 0.68 to 0.45, respectively) [50].

Therefore, we may think of pregnancy as a safe possibility for cancer survivors, but research on *BRCA*^mut^ women is still lacking, leaving a gap in our knowledge of the impact of pregnancy on this specific group of survivors. In recent years, Valentini and his team have attempted to shed some light on this field by enrolling a case group of *BRCA1/2* women diagnosed with BC while pregnant or who became pregnant after BC diagnosis, matching them with not pregnant–BC mutated carriers as controls. The 15-year survival rate was 91.5% for the “case group”, with a mean time of 2.4 years from diagnosis to gestation and 88.6% for the controls. Cases and controls were also free of recurrence at childbirth, and, respectively, 84.6% and 91.9% were still free after 15 years from diagnosis [49]. Recently, new data from Lambertini and colleagues confirmed no significant difference in disease-free survival between *BRCA*-mutated BC survivors with or without a pregnancy after cancer (0.81–1.20), enhancing how pregnancy should be regarded as a safe possibility and an essential aspect of survivorship care [51]

However, once pregnant, the decision to breastfeed can raise concerns about cancer risk since several studies demonstrated that in the general population, breastfeeding is associated with a lower risk of OC development. Recently, this correlation has been investigated by comparing *BRCA*^mut^ women between 18 and 80 yrs with clinical cancer to a control group of safe women with no pathological ovaries. A reduction of 23% in OC development risk was found in ever-feeding history than women who never breastfed. Furthermore, the protective effect increased from the 1st month to 7th, without further increase for more months and with any variation by the *BRCA* gene or age at diagnosis [52].

Despite this, breastfeeding choice is always personal and should be made together with healthcare equipment to provide individualized care based on the medical history and needs of each woman.

Actually, there are no specific screening guidelines in the case of pregnancy or breastfeeding in the presence of *BRCA* mutation, as well as optimal approaches for pregnancy and post-partum monitoring. Clearly, active surveillance would include semi-annual breast exams and imaging from 25 yrs to facilitate BC early detection, with mammography and MR being common options [18]; at least a periodic gynaecologic investigation for OC prevention should be included in *BRCA*^mut^ management.

## 5. The Impact of Chemotherapy on *BRCA*-Mutated Patients

Antineoplastic regimens used to treat cancer often have adverse effects on the reproductive system. Among them, alkylating agents, such as cyclophosphamide, are the most gonadotoxic cytostatics, capable of inducing apoptotic program in premature ovarian follicles and, consequently, leading to progressive follicle reserve depletion, named chemotherapy-associated ovarian failure (COF) [53]. Thus, since *BRCA*^mut^ is related to lower fertility, it has been suggested that they could be more affected by COF [54] compared to non-carriers. Oktay K. H. and coworkers confirmed this hypothesis and found that anti-Mullerian hormone (AMH) can be a reliable parameter to evaluate chemotherapy’s effect on the ovaries. Differently from older studies using amenorrhea [43], which can be a permanent or transient marker, AMH levels were tested in still-fertile breast cancer patients after treatment with doxorubicin, cyclophosphamide and paclitaxel. The recovery rate of ovarian function was observed to be lower in *BRCA*^mut^ (1.3%) compared to non-carriers (4.7%), indicating an accelerated ovarian aging process in mutated BC women [55].

However, Lambertini’s study provided conflicting data since no significant differences have been found between *BRCA*^mut^ and non-carriers (negative group) 1 year and 3 years after chemotherapy. In the *BRCA*-mutated group, AMH levels ranged from baseline concentrations of 1.94 µg/L to 0.09 µg/L after the first year and 0.25 µg/L after three yrs, while the non-mutated cohort started from 1.66 µg/L to 0.06 µg/L and 0.16 µg/L, respectively. The drastic decrease detectable after the first year of treatment is due to the cytotoxic effect of chemotherapy. In addition, *BRCA1* and *BRCA2* mutations showed a similar influence on measurement [56].

One of the most aggressive subtypes of BC is hormone receptor negative (HER2-). This subtype, also referred to as triple-negative breast cancer (TNBC), lacks estrogen receptor, progesterone receptor and human epidermal growth factor receptor 2 (HER2). About 60–70% of BC patients with an inherited *BRCA1/2* mutation fall into the TNBC subtype, and 10–30% of TNBC patients have a *BRCA* pathogenic variant [57].

In the presence of *BRCA1/2* LOF, poly(ADP-ribose) polymerase (PARP) acts as a backup system during the S phase to maintain the genome and repair accidental breaks at replication forks. PARP is an early response to single-stranded DNA break (SSB) and plays a crucial role in the base excision repair (BER) process. When BER fails to function properly, SSBs remain unrepaired, leading to the accumulation of damaged DNA and apoptosis. Consequently, treatment with PARP inhibitors greatly increases cell sensitivity to death. However, SSBs can also result in double-stranded DNA break (DSB) formation, which can be repaired through non-homologous end joining or homologous recombination. Therefore, inhibiting PARP alone is not sufficient to induce cell death, and TNBC patients still lack standardized treatment options [58].

Several clinical trials are still ongoing to verify whether PARP inhibitors can be used in combination with traditional neoadjuvant chemotherapy. However, the findings are controversial. At the same time, other studies are exploring the use of PARP inhibitors as a standalone treatment, such as Talazoparib [59].

Currently, few studies have discussed chemotherapy impairment on BRCA^+^ women’s fertility; thus, further studies are needed to identify reliable markers to evaluate ovarian function in this population undergoing cancer treatment.

## 6. Fertility Preservation Strategies for *BRCA* Mutation Carriers

When BRCA^+^ women cannot fulfill pregnancy before preventive surgery, assisted reproductive technology (ART) techniques can be considered.

The best-established FP option for *BRCA*-mutated women without cancer diagnosis is embryo/oocyte cryopreservation. They both consist of controlled ovarian hyperstimulation (COS), oocyte retrieval and cryostorage. Embryo cryopreservation is followed by in vitro fertilization (IVF) or intracytoplasmic sperm injection (ICIS) [60]. Ovarian preparation through COS takes about 2–5 weeks, in which patients receive exogenous recombinant follicle stimulation hormone (FSH), sometimes replaced with urinary FSH or administered in combination with gonadotropin-releasing hormone analogs (GnRHa) and aromatase inhibitors, such as letrozole or luteinizing hormone (LH). A potential iatrogenic controvert condition due to such hormone administration is ovarian hyperstimulation syndrome (OHSS). OHSS is described as increased capillary permeability leading to haemoconcentration, coagulation disorders or lethal pulmonary embolism, as well as a higher risk of cancer development, especially of hormone-sensitive ones, such as BC and OC [60].

Many theories have been proposed to explain the development of ovarian tumors probably related to IVF hormone stimulation protocol. Some of these hypotheses are the “continuous ovulation” theory, the trauma inflicted by oocyte retrieval, the combination of gonadotrophins with estrogens and growth factor receptors and others [61]. Gronwald and his team conducted the largest case-control evaluation on the potential link between infertility treatment and OC occurrence, requiring 941 BRCA carriers and controls [62]. Despite previous research suggesting that “incessant ovulation” protocols required for IVF may play a role in the development of OC, their findings revealed no significant statistical association, in agreement with previous studies conducted on general population risk [61].

When *BRCA*^mut^ was tested for ovarian response to COS, the last one was found to be lower with respect to *BRCA*-negative cohorts, with particular attention to *BRCA1*-mutated patients, who showed a 10-fold increased rate in comparison with the negative control group (33.3% vs. 3.3%) [63,64,65]. Different studies by Shapira M. and colleagues found instead no differences between the two groups in terms of oocytes collected, number of zygotes and fertilization rates [66,67].

If couples choose to undergo IVF procedures, they should be carefully informed concerning the possibility of preimplantation genetic diagnosis (PGD), also known as preimplantation genetic testing (PGT), which includes both diagnosis and screening.

PGD entails a single-cell extraction from a 6–8 cell embryo three days after the ICSI procedure, followed by polymerase chain reaction (PCR) for DNA analysis and research for the presence of a specific mutation before embryo implantation in the uterus. If the embryo is not affected by the investigated mutation and keeps growing, its transfer can be conducted on day 5 at the blastocyst stage [68]. According to ESHRE (European Society of Human Reproduction and Embryology) registries, PGT accounts for 5.9% of all accessible ART cycles in 40 European countries. Furthermore, PGT shows the steepest rise (+32.7%) in treatment numbers compared to previous registered results [69].

PGD aims to employ only healthy embryos for IVF procedures to avoid genetic disorder transmission to offspring; hence, it gains special value for patients with advanced age, recurrent miscarriages or prior failed IVF. Despite *BRCA* mutations being autosomal dominant, it is quite easy to reject embryos with respect to those investigated for recessive conditions, in which both alleles have to be compromised [70].

Even if embryo/oocyte cryopreservation is universally considered the first choice for FP, women carriers with already diagnosed neoplasia who cannot delay therapy, as well as prepubertal girls with no mature oocytes available, cannot access this practice [71]. Thus, ovarian tissue cryopreservation (OTC) may represent a valid alternative.

OTC involves the collection of ovarian cortex fragments and their orthotropic transplantation into the patient after oncological treatment, enabling the ovary’s endocrine function to be restored without treatment delay. Several studies reported that 85.2% of patients show endocrine function restoration, which endures for an average time of 26.9 months after ovarian tissue reimplantation [72].

However, before cryostorage, BRCA^+^ women tend to have lower oocyte numbers per mm^2^ (0.33 against 0.78) and per fragment (0.08 against 0.14) than in negative controls [73]. Despite this, ovarian function can be restored, as demonstrated by two cases of pregnancies carried out by young women without complications and with spontaneous deliveries and healthy child births [73,74]. Thus, as already affirmed by some clinicians [75,76], OTC should be definitively considered a non-experimental procedure.

**Figure 2:**
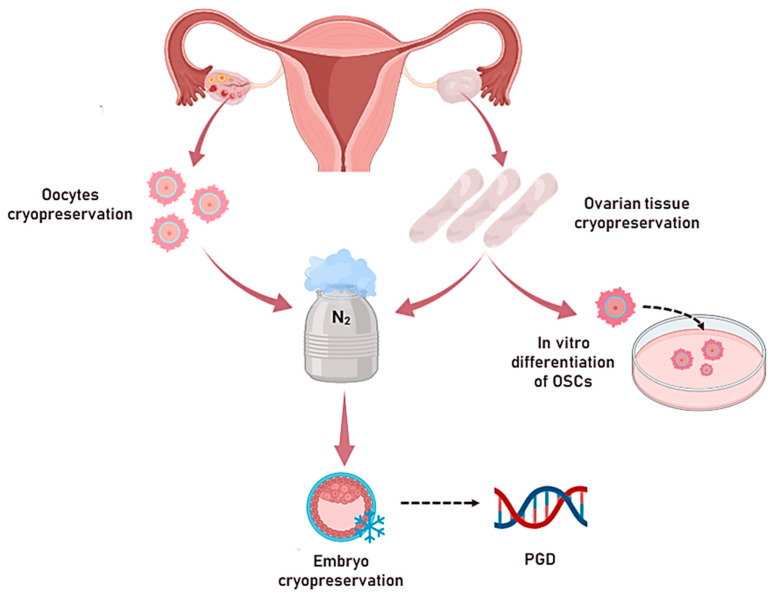
Fertility preservation (FP) techniques available for *BRCA*^mut^ carriers. The oocyte/embryo cryopreservation is a well-established procedure that ensures egg integrity of around 30/40% efficiency, although requiring hormonal stimulation with several related clinical effects on oncological patients. The ovarian cortex cryopreservation allows ovary endocrine function recovery and is the eligible practice for prepubertal patients, despite being experimental with a potential risk of cancer cell reimplantation after autologous graft. The ovarian stem cells (OSCs) isolated from the ovarian cortex own the ability to differentiate into mature oocytes in vitro (OLCs) under appropriate conditions. Despite this, the procedure is still experimental and requires an expert operator; it could, in the future, solve ethical problems related to the disposal of genetically abnormal embryos.

OTC is closely related to the field of ovarian stem cells (OSCs), which will be explained in more detail in the next paragraph. Figure 2 gives a resumption of the above-mentioned FP techniques.

## 7. OSCs: A New Frontier in Fertility Preservation for *BRCA* Carriers

In the field of reproductive medicine, stem cells represent an interesting issue, particularly in fertility preservation (FP). Among all stem cells, the most fascinating discovery in recent times has been the role of oogonial stem cells (OSCs), also known as germline stem cells. Experimental studies on mouse models already showed that OSCs could be a resource for “oocytes-like cells” (OLCs) [77] and could be employed for functional restoration of ovarian activity in cases of human OT grafting.

OSCs were first discovered in the surface epithelium of mouse ovaries, leading to the hypothesis that these cells could be identified in humans. This evidence has questioned the belief that women have a fixed oocyte number throughout their lifespan until OSCs were isolated from adult women’s ovarian cortex [77,78,79]; hence, oocyte production after birth may be possible. Stem cells, with their remarkable self-renewal, clonal expansion and differentiation abilities, can be harnessed to regenerate ovarian function and facilitate follicle generation. OSCs can be obtained from ovarian cortex enzymatic or mechanic digestion and plated to grow and replicate into stem clones, which then can be reintroduced in women at the end of cancer treatment to establish lost ovarian function and permit follicle generation.

Alternatively, it has already been demonstrated that OSCs can competently generate OLCs in vitro, providing a valuable resource for studying oocyte development and maturation. Recent studies reported that human OSCs can be identified through the surface expression of DEAD box polypeptide 4 (DDX4) germline marker, as well as FRAGILIS and STELLA, and other stemness molecules such as OCT-4 and stage-specific embryonic antigen 4 (SSEA-4) protein [80]. Once isolated and plated, proliferation stages occur and cell diameters keep increasing, moving from ~4 μm right after plating to ~20 μm after 1 week of culture to approximately 80–90 μm at complete differentiation [81]. As reported, under appropriate culture conditions, after 21 days, OSCs differentiate spontaneously into large haploid OLCs capable of entering miosis and expressing the major oocyte marker growth differentiation factor 9 (GDF-9) and synaptonemal complex protein 3 (SYCP3) [81,82,83]. Unfortunately, the identification of specific “oogenic” factors responsible for driving OSC differentiation is still under investigation; histone deacetylase inhibitors [84] and bone morphogenetic protein 4 (BMP4) have been proposed [85].

OSCs are still at the beginning of experimental employment, but they could be useful for solving ethical problems linked to PGD since the assessment of *BRCA*-negative conditions or other alterations implies single oocyte discard with respect to embryos, as in PGD.

Furthermore, it has already been demonstrated that OSCs can be obtained even from menopausal women [81,86] but, due to the inactivity of the ovulatory cycle, are probably unable to differentiate in vivo. In the future, this could represent an additional advantage for a wider approach to treating infertility, such as a heterologous graft.

## 8. Conclusions

*BRCA1* and *BRCA2* are tumor suppressor genes located on chromosome 17q21 and 13q12, respectively, and are responsible for an increased risk of breast and ovarian cancer development. Since they encode proteins involved in DNA double-strand break repair, mutations lead to the activation of apoptotic programs in cells. In ovaries, this mechanism is translated with loss of oocytes and, consequently, premature ovarian failure (POF), confirmed by lower anti-Mullerian hormone concentration compared to the general population.

In addition, the major susceptibility to chemotherapy of these patients in terms of ovarian insufficiency inevitably causes them to achieve pregnancy before the age of suggested prophylactic surgery.

Despite several studies on *BRCA* conveying limitations, such as small sample size, non-uniform cohort, environmental factor influence (i.e., smoking or diet) and age stratification, all of them reveal that these variants represent an additional trigger for cancer occurrence and fertility impairment. In this regard, personalized oncofertility counseling should be offered to all women at the diagnostic occurrence of *BRCA* mutations to help them make informed decisions about their fertility options.

Despite the challenges associated with *BRCA* mutations and fertility, there are many options available to women who wish to preserve their fertility. They include surgical RRSO, egg and embryo freezing or ovarian tissue cryopreservation. While these options can be expensive and time-consuming, they can help women with *BRCA* mutations achieve their motherhood desire. At last, ovarian stem cell isolation is a promising but still experimental technique that can avoid problems related to embryo discharge, such as in IVF.

Despite statistics analysis reflecting an increase in chosen FP options for *BRCA*-mutated patients, in the future, better efforts are necessary to guarantee a successful desire for motherhood in carriers.
